# Breast examination as a cost-effective screening tool in a clinical practice setting in Ibadan, Nigeria 

**DOI:** 10.4102/phcfm.v5i1.401

**Published:** 2013-02-14

**Authors:** Adetola M. Ogunbode, Akinola A. Fatiregun, Olayinka O. Ogunbode, Lawrence A. Adebusoye

**Affiliations:** 1 Department of Family Medicine, University College Hospital, Nigeria; 2 Department of Epidemiology and Medical Statistics, University of Ibadan, Nigeria; 3 Department of Obstetrics and Gynaecology, University of Ibadan, Nigeria

## Abstract

**Background:**

Breast cancer is a disease of public health importance. It results in high morbidity and mortality in women worldwide. The high morbidity and mortality from breast cancer can be decreased by measures targeted at early detection such as screening. Breast examination as a screening tool for breast cancer in developing countries is advocated in view of its cost-effectiveness.

**Method:**

The article selection method was obtained from primary and secondary literature sources which included original research articles, case control studies, review articles, proceedings, transactions and textbooks. The authors cited a clinical audit and articles published between 1988 and 2011. The search strategy included the use of internet search engines. This review was part of a larger research and the study protocol was approved by the University of Ibadan/University College Hospital, Ibadan Institutional Review Board (UI/UCH IRB). Clinical trial registration number-NHREC/05/01/2008a.

**Results:**

Breast self-examination (BSE) and clinical breast examination (CBE) as screening tools for breast cancer were analysed in detail.

**Conclusion:**

Breast examination is a screening tool that is cost-effective and reliable and should be encouraged in resource-constrained countries. Given the high cost and expertise required for mammography, current efforts at screening for breast cancer in developing countries should rely more on a combination of BSE and CBE.

## Abstrait

La palpation des seins comme outil de dépistage peu coûteux dans un contexte de centre médical à Ibadan, au Nigeria

**Contexte:**

Le cancer du sein est un important problème de santé publique. Celui-ci résulte sur une forte morbidité et mortalité chez les femmes dans le monde entier. La forte morbidité et mortalité associées au cancer du sein peuvent être réduites grâce à des mesures ciblées sur la détection précoce comme le dépistage. La palpation des seins comme outil de dépistage du cancer du sein dans les pays en développement est encouragée en raison de son faible coût.

**Méthodologie:**

La méthodologie de sélection de l’article découle de sources primaires et secondaires comprenant des publications scientifiques originales, des études cas témoin, des articles de synthèse, des discussions, des transactions et des manuels. Les auteurs citent une vérification clinique et des articles publiés entre 1988 et 2011. L’utilisation de moteurs de recherche sur Internet faisait partie de la stratégie de recherche. Ce rapport fait partie d’une étude plus étendue et le protocole d’étude a été approuve par l’université d’Ibadan/University College Hospital, l’Ibadan Institutional Review Board (UI/UCH IRB). Numéro d’enregistrement d’essai clinique -NHREC/05/01/2008a.

**Résultats:**

L’auto-palpation des seins (APS) et l’examen clinique des seins (ECS) comme outil de dépistage du cancer du sein ont été analysés en détail.

**Conclusion:**

L’examen des seins est un outil de dépistage peu coûteux, fiable et qui devrait être encouragé dans les pays à ressources limitées. Vu le coût élevé et l’expertise nécessaire pour la mammographie, les efforts actuels de dépistage du cancer du sein dans les pays en voie de développement doivent s’appuyer davantage sur une combinaison d’APS et d’ECS.

## Introduction

Cancer is a non-communicable disease, which is becoming increasingly important worldwide. It is a disease characterised by an abnormal growth of cells with the ability to invade adjacent tissues and even metastasize to distant organs, resulting in morbidity and eventually leading to the death of the individual if not detected and managed early. The aetiology is multifactorial and includes environmental factors such as tobacco smoking, tobacco chewing and alcohol consumption. Genetic factors are also implicated in the aetiology of cancer.^1^ 

Cancer affects all communities worldwide. Globally, about 10 million people are diagnosed with cancer and more than 6 million die of cancer every year.^1^ The burden of cancer is distributed unequally between developed and developing countries, with the highest incidence in affluent societies.^1^ In developing countries such as Nigeria, there appears to be a cancer epidemic similar to that in developed countries. Cancer is now becoming a burden on the health system and the economy as a whole. Any site of the body, including the breasts, can be affected by cancer. Breasts become prominent in female persons as the hallmark of puberty,^2^ with a breast made up of the axillary tail, lobules, the areola and the nipple. The breasts, modified sweat glands, are functionally of great importance for the offspring because the benefits of breastfeeding are manyfold and are dynamic structures that undergo changes throughout a woman’s reproductive life.^3^ 

Breast cancer is a disease of public health importance. Female malignancies such as breast cancer are an important aspect of the reproductive health problems of women and are a significant health problem for women worldwide. Reproductive health is defined as a state of physical and social well-being, and not merely the absence or infirmity, in all matters relating to the reproductive system and to its functions and processes.^4^ Breast cancer affects young, middle-aged, and elderly women who are caregivers of the family and who contribute to the development of society. Breast cancer is unfortunately still characterised by late presentation and poor outcome in many developing countries that lack the facility for early detection.^5^ It results in high morbidity and mortality which can be decreased by early detection and prompt treatment. 

Early detection of breast cancer can be accomplished through various screening methods and this is effective in reducing breast cancer mortality.^6^ Screening is the identification of individuals within an asymptomatic population who have, or are likely to develop, a specified disease at a time when intervention may prevent or halt the progression of the disease.^7^ In general, screening has two major objectives.^8^ One is the early detection of a disease at a point when treatment is more effective, less expensive, or both. Here, the implicit assumption underlying the concept of screening is that early detection, before the development of symptoms, will lead to a more favourable prognosis. This is because intervention initiated before the disease is clinically manifested will be more effective than treatment provided at a later stage of the disease. The second objective in screening is to identify risk factors that render an individual a higher than average risk for developing a disease, with the goal of modifying the risk factors to prevent or minimise the disease.^8^ Breast cancer definitely meets the above criteria for screening. It is surprising, however, that cancer of the breast still results in high morbidity and mortality despite the fact that the breast is an ‘exposed’ organ and is readily accessible to breast self-examination (BSE).^9^

### Epidemiology of breast cancer

The incidence of breast cancer varies in different parts of the world. The highest rates occur in the United States of America (USA) and Canada and the lowest rate is found in Asia.^10^ The incidence of breast cancer is increasing worldwide, but more rapidly in societies that hitherto enjoyed a low incidence of the disease such as most African countries.^11^ In the USA the incidence of breast cancer is 200 000 per annum with an annual mortality of about 40 000. In West Africa, despite a low regional incidence rate (27.8 per 100 000), the mortality rate of 19.6 per 100 000 is higher than in North America, which has an incidence rate of 99.4 per 100 000 and a mortality rate of 19.2 per 100 000. These differences between West Africa and North America could be a consequence of poverty, reduced public awareness, cultural beliefs and practices which contribute to women presenting late to health facilities in West Africa.^7^ Breast and cervical cancers are the two most common malignancies amongst Nigerian women, where they constitute about 50% of female cancers.^12^ In Nigeria, like in many developing countries, current reports have noted an increase in the incidence of breast cancer. The prevalence in Nigeria in 1976 was 15.3 per 100 000, but this had risen to 33.6 per 100 000 by 1992.^11^ This rise in prevalence could be a consequence of increased reporting and improved diagnostic methods.^7^ In addition, there is a trend towards westernisation in developing countries, with a change in the lifestyle, cultural habits, demographic and socio-economic profiles. 

Age is an important factor in the development of breast cancer. Breast cancer tend to occur in women after the age of 20 years, levelling up to a plateau at the age of 45–55 years, and thereafter increasing to a peak at 50–60 years.^2,13^ In developed countries, the age-specific incidence of breast cancer appears to increase with age with women in their fifties the most commonly affected. About 50% of breast cancers occur in the age group 50–65 years and 30% occur at an age older than 70 years.^3,7^ The peak age incidence of breast cancer is lower amongst Nigerian women (45–50 years) compared to European and American women where the peak age incidence was reported to be higher.^14^ Breast cancer has been found to be the leading cancer affecting young premenopausal and perimenopausal Nigerian women.^15^ Generally, breast cancer in Nigerian women presents a decade earlier than their counterparts in developed countries.^7^ It has a poor prognostic outlook, partly because a high proportion of patients presents with an advanced tumour as a consequence of non-recognition of the early signs of the disease by Nigerian women.^15^ 

## Methods

In a case control study of the epidemiological risk factors for breast cancer carried out at the Oncology clinic of University College Hospital (U.C.H), Ibadan, where 250 consecutive patients with breast cancer were recruited. These patients were individually matched by age with 250 patients with non-endocrine, non-malignant diseases admitted to the general surgical service during the same period. In this case control study, the epidemiological risk factors identified were age (mean patient age of 43 years), the use of oral contraceptives, nulliparity, a higher mean number of pregnancies, and heredity.^11^ 

### Prevention of breast cancer 

There are three levels of prevention of cancer.^16>^ Primary prevention targets the healthy population and includes preventive measures carried out at the family and community level. It involves health education about lifestyle modification issues and legislation.^1^ Secondary prevention comprises cancer registration, which could be hospital-based or population-based. It involves the early detection of cases through screening, as well as treatment.^1^ Tertiary prevention deals with the limitation of disability and with rehabilitation. 

### Breast cancer screening

Women need to be ‘breast aware’ and this can be accomplished through routine screening. By being ‘breast aware’, a woman is able to recognise symptoms of breast cancer through regular breast examination practice. At present the only demonstrable valid means of reducing morbidity and mortality because of breast cancer is by early detection and treatment.^13^ There exists, however, a wide range of modern procedures available for the screening and detection of breast cancer, such as the detection of the BRCA1 and BRCA2 genes, mammography, sonography, and recently, Magnetic Resonance Imaging (MRI) of the breast.^5^ Physical breast examination in the form of BSE and clinical breast examination CBE) are other screening methods used to detect breast cancer. BRCA1 and BRCA2 genes are breast cancer susceptibility genes which account for the majority of hereditary breast cancers.^9^ These are tumour suppressor genes, which are protective against the development of breast cancers.^9^ 

Mammography is a process whereby soft tissue x-rays are taken by placing the breast in direct contact with the films. Economic constraints in developing countries may impede the availability of mass-screening mammography, but its efficacy elsewhere is well-proven. Autier et al. (2011) stated in their report that the United Kingdom (UK) organised mammography screening programme was introduced in Northern Ireland at the beginning of the 1990s.^17^ It included women aged 50–64 years, who were invited to screening every 3 years. In 2003–2004, the age range was extended to women of up to 70 years. A national organised mammography screening programme was introduced in the Republic of Ireland in 2000, firstly in the eastern part of the country and then over several years it achieved nationwide coverage. Women aged 50–69 were invited every 2 years. Before that, breast screening was left to the discretion of the women and doctors and coverage was low. In 2002 national coverage was about 30%. It gradually increased and in 2008 it was 76%. From 1989 to 2006, breast cancer mortality decreased by 29.6% in Northern Ireland and by 26.7% in the Republic of Ireland.^17^ The most commonly used screening modality is mammography, but it is not readily available in most centres in developing economies.^5^ Mammography is very expensive and requires skilled personnel. It is, however, recognised that in developing countries, funding issues and lack of manpower are serious constraints to health care.^7^ Sonography is the use of the ultrasound equipment to examine the breast.^3^ Unfortunately these procedures, that is, mammography, sonography, and MRI of the breast, are expensive to implement in developing countries such as Nigeria. 

Despite the modern imaging screening technologies, CBE remains relevant as a screening tool in these resource-limited societies.^15^ In addition, in many developing countries such as Nigeria, BSE will most likely be the only feasible approach to wide population coverage because it is a cheap and practicable method.^14^ Even with newer screening methods, most cancers of the breast continue to be self-detected and this fact originally inspired the concept of physical breast examination, or BSE. Although the validity of this method has been in doubt, a number of studies has reinforced the validity of BSE in the detection of early breast cancer, and consequently in the reduction of morbidity and mortality from breast disease.^13^ Whereas BSE has become routine in developed countries, the situation in developing countries remains passive.^13^ Although there are suggestions that BSE may contribute to lower the risk of death through early detection of breast cancer, mammography has been shown to be a superior technique.^12^

### Physical breast examination

These include BSE and CBE. BSE is a process whereby women examine their breasts regularly to detect any abnormal swelling or lump in order to seek prompt medical attention. The American Cancer Society (A.C.S.) stated that ‘we must keep in mind the fact that at least 90% of the women who develop breast cancer discover the tumours themselves’.^18^ Furthermore, ‘training increases reported breast self-examination frequency, confidence, and the number of small tumours found’.^18^ There is a strong consensus that the effectiveness of BSE critically depends on careful training by skilled professionals, and that confidence in BSE is enhanced with annual CBEs by an experienced professional by using structured individual training.^18^ 

Unlike CBE and mammography, which require hospital visits, expertise and specialised equipment, BSE is inexpensive and can be carried out by the women themselves.^19^ BSE is an important, cheap and practicable method in the early diagnosis of breast cancer.^20^ Although there is controversy surrounding the efficacy of BSE in countries where CBE and mammography are readily available, elsewhere BSE remains a cost-effective method to detect breast cancer. A woman who performs regular BSE may be more motivated to seek medical attention, including CBE and mammography.^10^

### Technique and frequency of breast self-examination (BSE)

The BSE technique involves palpation of the breasts for lumps with the tips or pads of the fingers rather than with the flat of the hand. This could be performed whilst the woman is sitting down, standing or lying down. The purpose of a BSE is to learn the topography of the breasts, which in turn will allow one to notice changes in future so that breast masses or lumps can be detected. On the basis of the classifications of BSE frequency, the variability of BSE frequency may be reduced to three categories, (1) infrequent self-examination(performed hardly ever or not at all, once a year, or 3–4 times per year), (2) appropriate self-examination (performed monthly or fortnightly) and (3) excessive self-examination (performed weekly, daily, or more than once per day). 

It was found that higher levels of anxiety about breast cancer were associated with higher, rather than lower, BSE frequency. Concerns have been raised that anxiety experienced as a result of increased breast cancer risk may cause women to avoid screening. In fact, there are indications that women with a family history of breast cancer tend to perceive heightened personal susceptibility to breast cancer which may either promote compliance or deter them from practising BSE.^21^ BSE carried out once-monthly, between the 7th and the 10th day of the menstrual cycle, goes a long way to detecting breast cancer in the early stages of growth.^22^

### Prevalence and practice of breast self-examination (BSE)

In a cross-sectional, correlational study amongst 519 women from two major universities in Jordan, about 36% of the sample was university employees and 64% students (graduate as well as undergraduate).^23^ Stratified random sampling was used to enrol the undergraduate students, whereas the graduate students and employees were selected by convenience sampling. Although the majority of the sample population (67%) had heard or read about BSE, only a quarter of them reported that they had ever practised BSE in the previous 12 months, and only 7% had performed it on a regular monthly basis.^23^ 

In a cross-sectional study carried out amongst 406 female teachers in the Ilorin West Local Government area of Kwara State, Nigeria, in 2004, it was found that most (95.6%) respondents were aware of BSE.^22^ Despite the positive attitude of teachers to BSE, the practice of BSE was low (54.8%). One hundred and fifteen (71.8%) of the respondents who performed BSE, did it once a month; 12.5% indicated three times a month; 3.1% did it twice-yearly and 12.5% once a year.^22^ Of the 160 (49.0%) of the respondents who knew about BSE, 25 (15.6%) examined their breasts during the 1st week, 15 (9.3%) during the 2nd week, 18 (11.2%) in the middle of the cycle, and 102 (63.7%) at any period of the cycle, that is, irregularly. Fifty-five (34.3%) of the study population put a mark on a calendar as a reminder for the next BSE, whereas 105 (34.3%) did not.^22^ 

Nwagbo and Akpala in Enugu, Eastern Nigeria, in their study amongst 500 women within the child-bearing age of 15–44 years, found that only 62% of the women in the Enugu, Nigeria, practised BSE.^15^ The practice of BSE did not differ significantly amongst the various occupational groups in that study.^15^ Amongst 231 female traders recruited during a cross-sectional study in Ibadan, Oyo State, Nigeria, only 89 (37.1%) of the traders were aware of BSE and 51 (18.1%) of the respondents had ever practised BSE.^24^ Just 11 of the respondents (5.5%) had practised BSE in the report carried out amongst women seeking care in primary health-care centres in the Jos North Local Government area of Plateau state, Nigeria.^25^ In another study carried out in Nigeria amongst healthcare workers, it was reported that 89% of nurses practised BSE, even though the majority did not know the right time or technique for carrying out the procedure.^19^ A study conducted in the USA, showed that 89% of Afro-American women practised BSE in the past year, 74% in the past 6 months, and 39% indicated that they performed BSE on a monthly basis.^19^ 

A cross-sectional survey was conducted at the University of Ilorin and Kwara State Polytechnic, Ilorin in Kwara State Nigeria, amongst female students of the two institutions living in the hostels. Of the 704 students recruited, 700 responded. Even though more respondents (573 or 81.9%) had heard of breast self-examination, only 209 (29.8%) respondents claimed that they knew how to perform it.^26^ In Lagos University Teaching Hospital, Nigeria, in another cross-sectional study amongst female nursing students, only 135 of the 150 questionnaires handed out were collected and analysed. The respondents’ practice of BSE was good with 80.2% of the respondents claiming that they regularly performed BSE.^27^ 

Okolie, in a cross-sectional descriptive survey that assessed the knowledge, attitude and practice of BSE amongst 200 university female nursing undergraduates, between June and September 2011, found that the majority (92.35%) of the respondents had examined their breasts. Sixty-two (62%) of the respondents examined their breasts some days after menstruation, 32.14% did not have any particular time for examining their breasts, 4.08% examined their breasts during menstruation and 3.57% before menstruation. The majority (54.60%) examined their breasts anytime they felt like it; 33.67% did it once a month and 11.73% twice a month.^28^ Another descriptive hospital-based study was carried out amongst employees of two main health institutions in Bayelsa, Nigeria. Based on power calculations, 98 nurses were the legitimate sample size needed to participate in the study. Only 92, however, responded to the self-report questionnaire. Twenty-two (23.9%) practised BSE once a month and only 3 (3%) practised BSE more than once a month.^29^ 

### Clinical breast examination (CBE)

Physical examination of the breasts performed by skilled practitioners is called CBE. CBE is a screening method that requires only the physician’s fingers. The A.C.S. recommends that asymptomatic women over the age of 20 undergo regular CBEs as screening tests for breast cancer. CBE sensitivity is influenced by menopause, with the sensitivity found to be lower in premenopausal women.^30^ In postmenopausal breasts, there is more fat and less firm breast tissue, whereas in premenopausal breasts, there is less fat and more firm breast tissue. Obesity, race and hormonal use also influence CBE sensitivity.^30^ It was reported that CBE sensitivity decreased with increased body weight and that CBE was more sensitive in Asian women compared to white women.^30^ It was also documented that CBE sensitivities were higher amongst current versus non-current users of oestrogen and progesterone combination therapy.^30^ Some researchers have suggested that obese women and younger women receive less benefit from CBE.^31^ 

The critical importance and reliability of CBE was confirmed by the Canadian National Breast Cancer Screening Study.^18^ The study was a randomised controlled trial carried out on 40 000 women, aged 40–59 years on entry; these women were followed up by record linkage for 9–13 years, with active follow-up of cancer patients for an additional 3 years. Half the women performed monthly BSE, following instruction by trained nurses, had annual CBEs (duration 5 min–10 min) by trained nurses, and annual mammograms. The other half practised BSE and had annual CBEs but no mammograms.^18^ It was noted that in women aged 50–59 years, the addition of annual mammography screening to physical examination had no impact on breast cancer mortality.^18^ 

Ethnicity also affects performance of CBE. In a cross-sectional, random-digit-dialling telephone survey of 798 Latinos and 436 Anglos (non-Latino whites), it was reported that fewer (82.1%) Latino than Anglo women (88.9%) had CBE in the 2-year period of the study. It was concluded that Latino ethnicity is a relatively minor predictor of use of cancer screening tests, because these women were more likely to cite forgetfulness, lack of transportation, a long wait for appointments, and a need for child care as reasons for not having cancer screening tests.^32^ A descriptive study of 100 consecutive women at the antenatal clinic of a tertiary hospital in South-Eastern Nigeria showed that only 1% of the women had CBE performed by doctors, whereas 2% of them had CBE performed by nurses during the current antenatal period.^33^ The authors suggested that health-care providers should perform a thorough breast examination at the first prenatal visit and maintain a high index of suspicion for breast cancer throughout pregnancy.^33^ 

### Prevalence and practice of clinical breast examination (CBE)

Clinicians must use CBE to understand what patients find on their bodies, because a large percentage is found by the patients themselves.^34^ In addition, CBE by health professionals is still the only way that more than 10% of breast cancers are detected.^34^ Today, the most widely practised form of breast cancer screening is CBE. The prevalence of usage of CBE for breast cancer detection was 97% by all physicians and 99% by family practitioners in Illinois, USA.^12^ In a study, of 63 out of 82 family physicians from 4 out of the 6 health zones in Nigeria and the Federal Capital territory Abuja, Nigeria, it was found that CBE was carried out monthly by 48.9% of physicians on their regular patients.^12^ In another study by Okobia et al., in Nigeria, only 91 study participants (9.1%) had CBE in the past year. The main reasons given for not undergoing CBE included not having a breast problem (62.5%) and being unaware of the need for CBE (32.2%). None of the participants had ever had mammography screening performed.^19^ A cross-sectional survey conducted amongst nurses working in a general hospital in Lagos revealed that 204 nurses out of 280 participated in the study (73% response rate). Only 30%, however, had a clinical breast examination.^35^ 

Two hundred and twenty-eight students were selected by stratified random sampling from their different faculties in the University of Ibadan, Nigeria. Only 64 (28%) knew the interval for BSE, whereas 11 (25%) practiced BSE regularly.^36,37^ A multistage sampling technique was used to select two villages, and women from the Akinyele Local Government area of Ibadan, Nigeria, were interviewed. The assessment was performed with the use of a self-structured validated questionnaire administered by trained interviewers to 420 women randomly selected from the two health districts. Of the 43 women who had breast examination, 8 (2%) claimed to have been examined by health workers.^38^

### Characteristics and components of clinical breast examination (CBE)

A good clinical breast examination should include a comprehensive breast-related medical history to help establish possible risk factors such as personal and family history and a proper breast examination. There should be breast inspection for skin abnormalities such as skin puckering or dimpling, peau d’orange, ulceration and nipple asymmetry.^30^ In the report citing Baines and colleagues, the breast examination is performed with the woman seated or standing, firstly with arms relaxed and hanging, then with arms above the head, and finally with arms akimbo and hands pressing into the waist.^39^ The area to be examined should extend from the clavicle, medially to the mid-sternum, laterally to the mid-axillary line, and to the inferior portion of the breast. In addition, the examination should include the axillary tail of breast tissue and the axilla to search for possible lymphadenopathy. Palpation of the breasts should be performed with the woman in the upright (except for large-breasted women), supine, and oblique positions. The appropriate arm positions for the woman are for the upright posture, above the head; in the supine posture, firstly above the head for the medial breast examination and secondly at right angles to the body axis for the lateral breast examination; in the oblique position, ipsilateral-hand-to-forehead.^39^ 

CBE has several components, three of which had been systematically evaluated and found to influence the accuracy of the examination. These are the amount of time spent on the examination, the finger technique in palpation, and the search pattern utilised. First of all, clinicians should try to perform CBE in a deliberate fashion. Spending at least 2 min on the breast examination has been shown to improve sensitivity.^30^ By describing the findings to patients and reviewing the technique for BSE, and by conducting the CBE, women are provided with needed health education and feedback to understand what the clinician is evaluating. In addition, one of the most common reasons which women cite for failure to perform BSE is their inability to interpret physiologic nodularity. Patient education during the CBE had been shown to improve adherence to the BSE.^39^ 

The second important component of the CBE is the finger technique. This is carried out by using the pads of the second, third, and fourth fingers held together. The finger pads of the middle three fingers move in a dime-size circular motion applying three levels of pressure at each point, with light pressure initially, and then repeated in the same area with medium and deep pressure before moving to the next area to be examined.^30^ The third essential part of the CBE technique is the search pattern used to detect abnormalities. It is documented that a systematic search pattern that ensures that all breast tissue is examined, is essential for increasing the sensitivity of the clinical breast examination.^30^

There are three specific search patterns:

- The wedge or the radial spoke search pattern in which wedges of tissue are examined, beginning at the periphery and moving towards the nipple in a radial pattern.
- The second is the vertical strip or linear search pattern which examines the breast tissue in overlapping vertical strips across the chest wall.^39^ This pattern was developed by the National Cancer Institute, USA.
- The concentric circle or circular method is the third search pattern in which the breast is examined in larger or smaller concentric circles. This circular method is the most convenient and easiest to practise.

### Practical implications

The main contribution of this review paper is that breast examination as a screening tool can be used for early detection of breast cancer which in turn will help to reduce the mortality from breast cancer in resource-constrained countries. Physicians should be encouraged to perform CBE routinely in their clinical practice. Training of health workers and women in general on the breast examination technique could also be offered. 

### Limitations of the study

Like any other review article, this review is subject to bias such as the influence of the authors’ personal viewpoints, gaps in literature research that may lead to omission of relevant research, errors in translation of data from primary literature to summarisation in the review, misrepresentation or misinterpretation of original data sources. Efforts were made to minimise the influence of these potential sources of bias by considering the primary data thoroughly before examining the review items. Our understanding of the subject matter allowed us to critique the review articles and improve upon them. 

### Recommendations

Physicians should be encouraged to perform annual CBE routinely in their clinical practice. Training of other health workers on BSE methods should be carried out. Women should also be taught the breast examination technique.

### Conclusion

Breast examination as a screening tool should be used for early detection of breast cancer. This will help to reduce the mortality from breast cancer in resource-constrained countries. Its use should be encouraged in resource-constrained countries because it is affordable, reliable and practicable. Given the high cost of mammography screening and the skilled expertise required for the procedure, current efforts at breast cancer screening in Nigeria must rely on a combination of BSE and CBE. Excellent CBE performed by trained nurse-examiners may be as effective as mammography in reducing breast cancer mortality.^40^ Women can be taught the techniques of monthly BSE, and nurses, midwives, and other healthcare providers can be trained to augment physicians in the performance of CBE.^19^ 
